# Emotional Exhaustion and Job Satisfaction in Airport Security Officers – Work–Family Conflict as Mediator in the Job Demands–Resources Model

**DOI:** 10.3389/fpsyg.2016.00663

**Published:** 2016-05-09

**Authors:** Sophie Baeriswyl, Andreas Krause, Adrian Schwaninger

**Affiliations:** School of Applied Psychology, University of Applied Sciences and Arts Northwestern SwitzerlandOlten, Switzerland

**Keywords:** aviation security, JD–R model, supervisor support, work–family conflict, workload

## Abstract

The growing threat of terrorism has increased the importance of aviation security and the work of airport security officers (screeners). Nonetheless, airport security research has yet to focus on emotional exhaustion and job satisfaction as major determinants of screeners’ job performance. The present study bridges this research gap by applying the job demands–resources (JD–R) model and using work–family conflict (WFC) as an intervening variable to study relationships between work characteristics (workload and supervisor support), emotional exhaustion, and job satisfaction in 1,127 screeners at a European airport. Results of structural equation modeling revealed that (a) supervisor support as a major job resource predicted job satisfaction among screeners; (b) workload as a major job demand predicted their emotional exhaustion; and (c) WFC proved to be a promising extension to the JD–R model that partially mediated the impact of supervisor support and workload on job satisfaction and emotional exhaustion. Theoretical and practical implications are discussed.

## Introduction

Since September 11, 2001, security checks at airports have become vitally important, and strict security controls based on advanced technology are implemented to minimize the threat of terrorism. One major aspect in the airport security process is hand luggage and passenger controls with x-ray machines (for a recent review, see Wetter, 2013). Before entering the secure area of an airport, all passengers, airline staff, and airport staff have to pass a security checkpoint at which they and all their belongings are subjected to strict controls. By guaranteeing a smooth procedure at security checkpoints when inspecting passenger luggage with x-ray imaging and by carrying out follow-up checks on passengers and hand luggage, airport security officers (screeners) perform vital security tasks. Studies with screeners have seen an emphasis on visual search in x-ray images, optimizing technology, and using security training programs to improve detection performance (e.g., von Bastian et al., 2008; Koller et al., 2009; Halbherr et al., 2013; Mendes et al., 2013; Wolfe et al., 2013; Biggs and Mitroff, 2014; Mitroff et al., 2015). Up to now, the health and well-being of screeners have not been the focus of research. This study addresses this research gap by investigating important variables of health and well-being in 1,127 screeners at a European airport.

### Burnout and Job Satisfaction

Burnout and job satisfaction rank among the most frequently used indicators of mental health and well-being in work and organizational psychology. Burnout is a tripartite syndrome consisting of emotional exhaustion, feelings of depersonalization (also named cynicism), and awareness of reduced personal accomplishment (Maslach, 1982). Emotional exhaustion is characterized by feelings of mental fatigue or of being emotionally drained. Depersonalization is marked by a detached and emotionally distanced treatment of clients, co-workers, and the organization. And finally, diminished personal accomplishment is characterized by a negative evaluation of one’s job competence and effectiveness. Emotional exhaustion is most often seen as the key component of burnout (Cordes and Dougherty, 1993; Lee and Ashforth, 1993; Cropanzano et al., 2003) and it has been associated with diverse negative organizational outcomes and personal dysfunctions such as an increased prevalence of counterproductive work behavior, turnover intentions, and sickness absence as well as mental health problems (Cordes and Dougherty, 1993; Lee and Ashforth, 1996; Borritz et al., 2006; Bolton et al., 2012). Job satisfaction is one of the most broadly studied variables in work and organizational psychology (Dormann and Zapf, 2001) and is commonly defined as a “pleasurable or positive emotional state resulting from the appraisal of one’s job or job experiences” (Locke, 1976, p. 1304). It can refer to a global evaluation of one’s job situation or to the evaluation of individual facets of everyday work (i.e., satisfaction with work itself, supervision, coworkers, pay, and promotional opportunities) (e.g., Ironson et al., 1989). Job satisfaction has been shown to be associated with various organizational and individual outcomes. For example, high levels of job satisfaction have been associated negatively with intent to leave one’s job (Hellman, 1997; Fried et al., 2008) and positively with life satisfaction and happiness (Bowling et al., 2010).

Emotional exhaustion and job satisfaction have also been proven to be highly relevant factors for performance in a wide range of organizational settings: emotional exhaustion in the sense of an inhibitor of good job performance (Parker and Kulik, 1995; Wright and Bonett, 1997; Wright and Cropanzano, 1998; Cropanzano et al., 2003; Bakker et al., 2004) and job satisfaction in the sense of a promoter of good job performance (Judge et al., 2001; Wright et al., 2007). Moreover, empirical studies have discussed burnout and job satisfaction as major determinants of safety performance (Siu et al., 2004; Nahrgang et al., 2011). This most certainly also applies to the performance of screeners and thus to the security concept at airports; and it clearly reveals the importance of identifying the antecedents of emotional exhaustion and job satisfaction in this important occupational group.

### The Job Demands–Resources (JD–R) Model

The JD–R model is a well–tested and widely used theoretical framework for describing the relationships between work characteristics and well–being. The model includes aspects from various theories and is thus broader and more comprehensive than older models such as the demand–control model (Karasek, 1979) or the effort–reward-imbalance model (Siegrist, 1996). Moreover, it contains assumptions taken from the job characteristics model (Hackman and Oldham, 1976) and the model of conservation of resources (Hobfoll, 1989) by underlining that job resources are not only necessary to deal with high job demands but also important in their own right (Bakker and Demerouti, 2007). The central proposition of the JD–R model is the assumption of dual processes. Within the first, the *health impairment process*, high job demands exhaust the employees’ mental and physical resources and can subsequently lead to ill-health. Job demands refer to “physical, psychological, social, or organizational aspects of the job that require sustained physical and/or psychological (cognitive and emotional) effort or skills” (Bakker and Demerouti, 2007, p. 312). Hence, they can be associated with physiological and/or psychological costs. Typically, emotional exhaustion is used as an indicator of poor mental health in the model. Although numerous job demands have been identified as predictors of emotional exhaustion (Cordes and Dougherty, 1993), meta-analyses have confirmed that the key determinants of emotional exhaustion in various occupational settings are workload and associated phenomena such as time or work pressure (Lee and Ashforth, 1996; Alarcon, 2011; Bowling et al., 2015). In light of current trends in air traffic toward major growth in the quantity of passengers and luggage accompanied by staff cuts due to cost pressure, workload appears to be an important feature of the screeners’ working environment that is of potential relevance for their health and well-being (Karimbocus, 2015). And the workload will get even higher, given the estimation of an additional 4 billion passengers flying per year within the next 20 years (Benda, 2015). Nonetheless, to the best of our knowledge, workload and its effect on emotional exhaustion have not yet been examined in security staff in general and in screeners in particular. Based on both the JD–R model and the empirical findings stated above, we expect workload to be positively related to emotional exhaustion in our sample of screeners (Hypothesis 1).

The second process in the JD–R model is *motivational* in nature. Job resources are assumed to be key requirements for internal and external motivation because they support the satisfaction of basic needs and the achievement of work goals. Job resources describe “physical, psychological, social, or organizational aspects of the job that are functional in achieving work goals, reduce job demands and the associated physiological and psychological costs, and/or stimulate personal growth, learning, and development” (Bakker and Demerouti, 2007, p. 312). Job resources are considered to be central determinants of positive motivational states such as high work engagement and organizational commitment (e.g., Schaufeli et al., 2009). Despite being one of the most important and widely researched variables in industrial and organizational psychology (Dormann and Zapf, 2001), job satisfaction is not a widely acknowledged factor in the JD–R model. Nonetheless, a few studies have considered the relationship between job resources and job satisfaction against a JD–R background (Lewig and Dollard, 2003; Angulo and Osca, 2012; Biggs et al., 2014). Comprehensive research has shown that workplace social support is an important condition conductive to job satisfaction (Locke, 1976). Social support has been defined broadly as “the availability of helping relationships and the quality of those relationships” (Leavy, 1983, p. 5). However, a global definition does not take into account the complexity of social support, because it can stem from different sources (e.g., Baruch-Feldman et al., 2002). One common source of workplace social support is the supervisor. Numerous studies have revealed that the availability of supervisor support is associated with higher job satisfaction (e.g., Lewig and Dollard, 2003; Cortese et al., 2010; Biggs et al., 2014). Supervisor support might be particularly important for a screeners job satisfaction, because other typically discussed job resources (e.g., autonomy) and other sources of support (e.g., coworkers, customers) are typically available to a limited extent in this work field: detailed standard operating procedures result in less flexibility for screeners to use their professional decision-making skills and constrain their scope of action. Moreover, screeners work in varying work teams and perform rather uncomfortable tasks in relation to passengers. This does not help them to gain social support and recognition from coworkers and customers. Accordingly, we expect supervisor support to be particularly important for airport screeners and to be a good predictor of their job satisfaction (Hypothesis 2).

### Work–Family Conflict as an Intervening Variable in the JD–R Model

The JD–R model has undergone several extensions in the past few years by integrating, for example, personal resources and the concept of job crafting (Xanthopoulou et al., 2007; Petrou et al., 2012) or by examining the accumulative effect of different job demands (van Woerkom et al., 2016). However, due to the parsimony of the JD–R model, there are still open questions regarding not only the processes leading to health impairment such as emotional exhaustion but also the motivational outcomes such as job satisfaction (Demerouti and Bakker, 2011; Fernet et al., 2013). The increasing number of dual career couples has raised the importance of the (in) compatibility of family and work roles in predicting employees’ health and well-being (Frye and Breaugh, 2004). Moreover, the work–family interface can be expected to be of particular importance among screeners, because they work in changing shifts, a working condition that has turned out to be relevant in terms of the compatibility of family and work (Beutell, 2010).

Several models have been advanced to explain the relationship between work and family roles (Guest, 2002; Voydanoff, 2002). The segmentation model hypothesizes that work and family are two distinct domains of life that have no influence on each other. The spillover model, in contrast, hypothesizes that one domain can influence the other in either a positive or negative way. The kind of influence in the context of spillover theory can be considered from three perspectives: (1) domains can influence each other either within (classical spillover perspective) or between (crossover perspective) individuals (Bakker et al., 2008; Pedersen and Minnotte, 2012); (2) effects from one domain to the other can be either positive (enrichment, facilitation) or negative (conflict) (e.g., Carlson et al., 2006; Innstrand et al., 2008; Odle-Dusseau et al., 2012); and (3) effects can take either the direction work → family or family → work (Frone et al., 1992; Innstrand et al., 2008; Odle-Dusseau et al., 2012). In the present study, we focus on negative spillover from work to family within the individual and subsequently name it work–family conflict (WFC). Comprehensive research has demonstrated the effect of WFC on health and well-being (e.g., Allen et al., 2000; Amstad et al., 2011). WFC can, on the one hand, foster emotional exhaustion (Demerouti et al., 2004, 2005; Hall et al., 2010; Karatepe, 2010) and, on the other hand, hamper aspects of overall well-being such as job satisfaction (Frye and Breaugh, 2004; Karatepe and Kilic, 2007; Cortese et al., 2010; Beutell and Schneer, 2014). Although a number of job demands and job resources have been identified as determinants of WFC, workload and social support have emerged as the major antecedents in the sense that greater workload increases WFC and greater supervisor support reduces WFC (Byron, 2005; Michel et al., 2011; Bowling et al., 2015). In the context of supervisor support, both supervisor work–family support (Frye and Breaugh, 2004; Yildirim and Aycan, 2008; Lu et al., 2015) and more global ways of supervisor support (in the sense of emotional and/or instrumental support) (Thompson et al., 2006; Karatepe and Kilic, 2007) have been shown to reduce WFC.

Several theoretical frameworks have been used to guide the study of WFC. Popular theories are the role stress theory (Kahn et al., 1964), the conservation of resources theory (Hobfoll, 1989), and the JD–R model (Bakker and Demerouti, 2007). Nonetheless, WFC has been conceptualized differently and, consequently, has been located in the stressor–strain chain as either an independent (e.g., Schaufeli et al., 2009; Guglielmi et al., 2012), dependent (e.g., Bakker and Geurts, 2004; Boyar et al., 2014), or intervening variable (e.g., Peeters et al., 2004, 2005). Following the recommendation of Peeters et al. (2004) to distinguish the concept clearly from other job demands, we decided to view WFC as an intervening variable and integrate it as such into the JD–R model as our theoretical framework. WFC has been shown to mediate the positive effects of workload on emotional exhaustion (Peeters et al., 2004; Demerouti et al., 2005; Hall et al., 2010). High workload can cause a depletion of resources (Bakker and Demerouti, 2007). If the corresponding resources are no longer available in private life, this can be a reason for WFC (cf. Semmer et al., 2010). WFC, in turn, can amplify the experienced demands and subsequently lead to emotional exhaustion through impaired recovery (Geurts et al., 2003). However, WFC may not be the only mechanism linking workload with emotional exhaustion. For example, coping may be an alternative mediating mechanism: the study of Snow et al. (2003) revealed that work stressors increased avoidance coping which, in turn, increased strain. Therefore, we expect the effect of workload on emotional exhaustion to be partially mediated by WFC (Hypothesis 3a).

Evidence related to the indirect effect of supervisor support on job satisfaction is inconsistent. A great number of studies have revealed that supervisor support is related negatively to WFC, and that this, in turn, decreases job satisfaction (Frye and Breaugh, 2004; Thompson et al., 2006; Karatepe and Kilic, 2007; Yildirim and Aycan, 2008; Cortese et al., 2010; Lu et al., 2015). However, recent empirical findings have revealed non-significant indirect effects (Ito and Brotheridge, 2012; Odle-Dusseau et al., 2012). The present study contributes to gaining a better understanding of supervisor support by examining it in a sample of screeners. As predicted by the JD–R model, resources available in the work domain may energize the motivational process, and this may then facilitate better adjustment and consequently reduce WFC (Lu et al., 2015). Reduced WFC makes it possible to direct personal resources toward attaining work goals, and this, in turn, promotes job satisfaction. Family-friendly support by supervisors could be particularly important for screeners, because they have to work in changing shifts. Supervisors may support a family-friendly organization of work—either directly through approving family-friendly initiatives (Frye and Breaugh, 2004; Yildirim and Aycan, 2008; Lu et al., 2015) or indirectly through scheduling family-friendly shifts (Beutell, 2010). This, in turn, may well enhance commitment and satisfaction among screeners. We expect the effect of supervisor support on job satisfaction to be partially mediated by WFC in our sample of screeners (Hypothesis 3b), because other mediating mechanisms such as coping may exist (cf. Snow et al., 2003). In addition, it can be expected that job resources such as supervisor support satisfy basic needs and values and, thus, foster job satisfaction also directly (Bakker and Demerouti, 2007).

**FIGURE 1:**
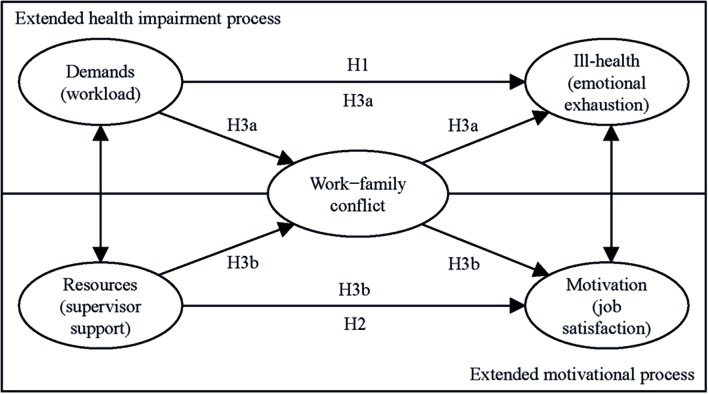
**Extended version of the JD–R model.** H, Hypothesis.

**Figure 1** presents the extended version of the JD–R model and an overview of our hypotheses. Our study of emotional exhaustion, job satisfaction, and WFC goes beyond previous research because we analyze the effects of workload and supervisor support on WFC, emotional exhaustion, and job satisfaction in screeners—a population in which health and motivational variables have not been the focus of research so far, despite their substantial role in aviation security. Preventing emotional exhaustion and promoting job satisfaction among screeners is not just important for its own sake but also in view of the need to protect airports and air travelers, thereby making it a matter of strong public interest. Additionally, our study broadens recent WFC research by simultaneously integrating the concept as an intervening variable into the health impairment and the motivational process of the JD–R model. As a results, it contributes to gaining a better understanding of the indirect effect (mediated via WFC) of supervisor support on job satisfaction.

## Materials and Methods

### Participants and Procedures

We used a cross-sectional analysis to survey screeners employed at a European airport in a German-speaking country. The survey took the form of a paper-and-pencil questionnaire that operative leaders distributed to the screeners in their group during the morning briefing. Screeners absent for longer periods (e.g., due to disability or illness) received the questionnaire by mail. This study was carried out in accordance with the Declaration of Helsinki and data protection regulations of the relevant country. The study was approved by the Work Council of the respective airport and subjects gave written informed consent. From a total of 2,166 distributed questionnaires, 1,329 screeners participated in the survey. In seven cases, the amount of missing data was above 30%. These questionnaires were excluded from further analysis (cf. Hair et al., 1998). This left data from 1,322 screeners that were suitable for statistical analyses–a response rate of 61%. Because the work of screeners inspecting *hold baggage* (check-in luggage) is different^1^ to that of screeners at the checkpoints (inspecting *carry-on cabin baggage*), we excluded 153 screeners who partly or predominantly inspected hold baggage and 42 screeners who did not state the kind of work they performed most often. This left a final sample of 1,127 screeners.

We investigated six demographic variables: gender (1 = female; 2 = male), age (1 = 29 years and younger; 2 = 30–39 years; 3 = 40–49 years; 4 = 50 years and older), job tenure (1 = 2 years and less; 2 = 3–6 years; 3 = 7–11 years; 4 = 12 years and more), marital status (1 = in partnership; 2 = not in partnership), children of preschool age, and children of school age (1 = yes; 2 = no). The sample contained 496 (44%) female and 620 (55%) male respondents. Eleven participants (1%) did not state their gender. A total of 1,115 participants (99%) reported their age. Most were aged 40–49 years (359, 32%); 316 (28%) were aged 50 years or older; and 315 (28%) were aged 30–39 years. The remaining 125 respondents (11%) were 29 years old or younger. Most participants reported long job tenure. A total of 372 (33%) had been employed in the company for 7–11 years; 355 (32%), for 12 years or longer; 173 (15%), for 3–6 years; and 207 (18%), for 2 years or less. The remaining 20 participants (2%) did not report job tenure. Most respondents (801, 71%) were in a partnership at the time of the survey, 298 (26%) were single, and 28 (3%) did not state their marital status. Regarding children, 165 respondents (15%) had children of preschool age and 932 (83%) did not; 307 (27%) had children of school age and 796 (71%) did not; and 30 respondents (3%), respectively, 24 respondents (2%) did not answer the respective question about children. In addition to the demographic variables, we assessed the conditions of employment. We asked participants whether they worked full or part time (1 = full time; 2 = part time) and about the type of shift work they usually performed (1 = changing shifts; 2 = fixed shifts or day duty). The majority of respondents (897, 80%) worked full time, 220 (19%) worked part time, and 10 participants (1%) did not report whether they worked full or part time. Just over one-half (606, 54%) worked predominantly in changing shifts; the remaining participants worked either fixed shifts or day duty (475, 42%) or did not state their duty (46, 4%).

Based on operating data from the company, we were able to estimate the representative status of our sample. The sample of screeners was representative (in terms of demographic characteristics and conditions of employment) of the entire group of screeners at the airport with two exceptions: first, older screeners were slightly overrepresented, whereas young screeners were slightly underrepresented. Second, screeners with little work experience in the company (2 years or less) were also slightly underrepresented. These data related to the entire sample of screeners including an occupational group not included in our analyses (i.e., 217 screeners inspecting *hold baggage*). However, we did not expect this occupational group to differ in terms of demographics and employment characteristics from the screeners at the checkpoints.

### Measures

We assessed workload with a single item taken from the German version (Rödel et al., 2004) of Siegrist et al.’s (2004) effort scale (“I have constant time pressure due to a heavy workload.”) that was rated on a 4-point scale ranging from 1 (*strongly disagree*) to 4 (*strongly agree*).

To assess supervisor support, we used the corresponding scale from the *Salutogenetische Subjektive Arbeitsanalyse* [salutogenetic subjective work analysis] (SALSA; Rimann and Udris, 1997). This scale measures the perceived availability of emotional and instrumental support from the supervisor and contains three items (e.g., “To what extent is your supervisor willing to listen to your problems at work?”). We used the original 5-point scale ranging from 1 (*not at all*) to 5 (*absolutely*). Cronbach’s α was 0.85.

Emotional exhaustion was measured with the personal burnout scale (Nübling et al., 2006, based on Borritz and Kristensen, 1999). The scale has six items (e.g., “How often are you emotionally exhausted?”) that were rated on the original 5-point response scale ranging from 1 (*never*) to 5 (*always*). Despite the label personal burnout, the corresponding scale measures the emotional exhaustion component of burnout. Cronbach’s α was 0.92.

We assessed job satisfaction with five items (e.g., “I enjoy my work.”) from the *Diagnose gesundheitsförderlicher Arbeit* [diagnosis of health-promoting work] (DigA; Ducki, 2000). These measure global job satisfaction on 5-point scales ranging from 1 (*not true*) to 5 (*absolutely true*). Cronbach’s α was 0.86.

Finally, we used a German translation (Nübling et al., 2006) of the work–privacy conflict scale (Netemeyer et al., 1996) as an indicator of WFC. The scale has five items (e.g., “The demands at my work interfere with my home life.”) rated on a 5-point scale ranging from 1 (*disagree*) to 5 (*totally agree*). Cronbach’s α was 91. Note that this scale measures solely work-to-family conflict (and not family-to-work conflict).

### Data Analysis

We examined reliability (Cronbach’s α), descriptive statistics (*M* and *SD*), and correlations (Pearson product–moment and point-biserial correlations, Spearman correlations, Phi coefficient, Cramér’s *V*) in SPSS Version 22. A Cronbach’s α of 0.70 or higher can be rated as adequate for the present analysis (Nunnally and Bernstein, 1994). We recoded items so that high values reflected a high level of workload, supervisor support, WFC, emotional exhaustion, and job satisfaction.

We performed confirmatory factor analyses (CFA) and multivariate analyses with *structural equation modeling* (SEM) in Amos Version 22 using maximum likelihood (ML) methods of estimation. We chose this analytical technique because of its strengths in terms of controlling for measurement error while simultaneously considering multiple predictors and outcomes as well as reliably measuring relevant constructs through the aggregation of multiple indicators (Kline, 2011). In CFA, factor loadings should be at least 0.50 and ideally 0.70 or higher (Hair et al., 2014). To estimate the global fit of the models we used the χ^2^ value. However, the χ^2^ value depends largely on sample size: in models based on large samples, the χ^2^ value is high and mostly significant (thus indicating a poor fit). Because our sample was quite large, we used two alternative measures to assess global model fit: the root-mean-square error of approximation (RMSEA) and the comparative fit index (CFI). RMSEA values up to 0.08 (Browne and Cudeck, 1993) and CFI values close to 0.95 (Hu and Bentler, 1999) indicate a good fit between the proposed model and the data.

To test Hypotheses 1 and 2, we computed a basic Model M_0_ with workload and support as predictors of emotional exhaustion and job satisfaction without WFC as intervening variable. Recent empirical evidence has indicated the presence of crossover effects (i.e., effects of demands on motivational outcomes and effects of resources on health-related outcomes) in the JD–R model (Knudsen et al., 2009; Crawford et al., 2010; Li et al., 2013; Ângelo and Chambel, 2014). Therefore, we integrated these effects in M_0_ along with the effects assumed in Hypotheses 1 and 2. To test for the proposed partial mediation effects assumed in Hypothesis 3, we then entered WFC as intervening variable into Model M_1_. This included both direct paths from workload and support to emotional exhaustion and job satisfaction and indirect paths mediated via WFC. Again, the model allowed for crossover effects. We tested the significance of path coefficients with the bootstrapping method and imputed missing data with the regression method. We considered ML estimates of the indirect effects and bootstrap confidence intervals (cf. MacKinnon et al., 2007).

We integrated relevant demographic variables and conditions of employment as control variables into the final model and considered their potential effects on model parameters.

## Results

### Descriptive Statistics and Correlations

**Table 1** presents means and standard deviations of the study variables and correlations between the study variables, demographic variables, and conditions of employment. Workload related negatively to supervisor support. Workload related positively to emotional exhaustion and WFC, but was unrelated to job satisfaction. Supervisor support related negatively to WFC and emotional exhaustion and positively to job satisfaction. Emotional exhaustion and job satisfaction interrelated negatively, and the former had a positive and the latter a negative relation to WFC.

**Table 1:** Descriptive statistics and correlations (Pearson, Spearman, Phi coefficient, Cramér’s *V*) between study variables (*N* = 1,127).

	*M*	*SD*	1	2	3	4	5	6	7	8	9	10	11	12	13
1. Workload	3.30	0.81	-												
2. Supervisor support	2.53	0.88	-0.17^∗∗∗^	-											
3. Work–family conflict	3.75	0.99	0.29^∗∗∗^	-0.22^∗∗∗^	-										
4. Emotional exhaustion	3.40	0.80	0.40^∗∗∗^	-0.27^∗∗∗^	0.61^∗∗∗^	-									
5. Job satisfaction	2.74	0.93	-0.06	0.29^∗∗∗^	-0.25^∗∗∗^	-0.37^∗∗∗^	-								
6. Gender^1^	1.56	0.50	-0.07^∗^	0.05	0.06^∗^	-0.06^∗^	-0.03	-							
7. Age^2^	2.78	0.98	0.08^∗∗^	0.05	-0.05	-0.02	-0.11^∗∗∗^	0.05	-						
8. Job tenure^3^	2.79	1.09	0.16^∗∗∗^	-0.09^∗∗^	-0.09^∗∗^	0.12^∗∗∗^	-0.26^∗∗∗^	0.10^∗^	0.45^∗∗∗^	-					
9. Marital status^4^	1.27	0.45	-0.01	0.04	0.05	0.00	0.01	-0.03	0.13^∗∗∗^	0.15^∗∗∗^	-				
10. Children of preschool age^5^	1.85	0.36	0.08^∗∗^	0.11^∗∗∗^	0.04	0.02	-0.03	-0.08^∗∗^	0.24^∗∗∗^	0.07	0.15^∗∗∗^	-			
11. Children of school age^6^	1.72	0.45	0.01	0.03	0.07^∗^	0.03	-0.03	-0.08^∗^	0.28^∗∗∗^	0.17^∗∗∗^	0.21^∗∗∗^	0.33^∗∗∗^	-		
12. Working full/part time^7^	1.20	0.40	0.04	0.02	-0.15^∗∗∗^	-0.03	-0.05	-0.25^∗∗∗^	0.11^∗∗^	0.14^∗∗∗^	-0.04	-0.03	-0.12^∗∗∗^	-	
13. Shift^8^	1.44	0.50	0.03	0.02	-0.15^∗∗∗^	-0.03	0.03	-0.17^∗∗∗^	0.07	0.21^∗∗∗^	-0.01	-0.05	-0.04	0.32^∗∗∗^	-

*^1^1 = female; 2 = male. ^2^1 = ≤ 29 years; 2 = 30–39 years; 3 = 40–49 years; 4 = ≥ 50 years. ^3^1 = ≤ 2 years; 2 = 3–6 years; 3 = 7–11 years; 4 = ≥ 12 years. ^4^1 = in partnership; 2 = not in partnership. ^5^1 = yes; 2 = no. ^6^1 = yes; 2 = no. ^7^1 = full time; 2 = part time. ^8^1 = changing shifts; 2 = fixed shifts or day duty. ^∗^p ≤ 0.05, ^∗∗^p ≤ 0.01, ^∗∗∗^p ≤ 0.001.*

Demographic variables and employment characteristics related to the study variables only to a minor degree. The correlation coefficients attained only partial significance and effect sizes were typically small (Cohen, 1988). Only job tenure showed some considerable relationships with our study variables, namely with workload, emotional exhaustion, and job satisfaction: participants with longer job tenure perceived a higher workload and they also reported higher emotional exhaustion and lower job satisfaction. Additionally, respondents with changing shifts and respondents with full-time work arrangements reported higher WFC. We therefore integrated the variables job tenure, working full/part time, and shift as control variables into the final Model M_1_.

### Results of Structural Equation Modeling

We constructed the latent variables supervisor support, emotional exhaustion, job satisfaction, and WFC on the basis of several observed items. CFAs revealed that the integrated constructs were of good quality: all indicators showed statistically significant factor loadings (*p* < 0.01 or *p* < 0.05). These were clearly higher than the quality criterion of 0.50 recommended by Hair et al. (2014). Moreover, most of the factor loadings were close to or higher than 0.70, indicating that the constructs were of a very good quality. We used the single item of workload as an observed variable.

**FIGURE 2:**
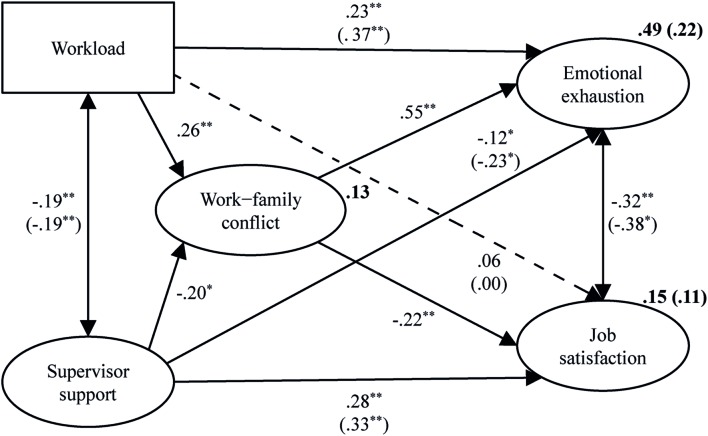
**Maximum likelihood (ML) estimates for Model M_1_ (*N* = 1,127).** The standardized regression weights of Model M_0_ are depicted in parentheses. The broken line represents a non-significant effect. Amounts of explained variance are depicted in bold print. Workload is represented by a rectangle, because the single item was integrated into the model as a manifest variable. The standardized indirect effects of workload on emotional exhaustion (0.14, *p* = 0.006) and supervisor support on job satisfaction (0.05, *p* = 0.009) were statistically significant. ^∗^*p* ≤ 0.05, ^∗∗^*p* ≤ 0.01.

To test Hypotheses 1 and 2, we first considered Model M_0_ without WFC as intervening variable. As Amos does not allow correlating the two endogenous variables, we correlated emotional exhaustion and job satisfaction through their error terms. Model M_0_ fitted the data well, χ^2^(85) = 465.71, *p* = 0.000, CFI = 0.96, RMSEA = 0.06. The standardized regression weights and their significance are shown in parentheses in **Figure 2**. Higher levels of workload were associated significantly with higher levels of emotional exhaustion. This supported Hypothesis 1. Higher levels of supervisor support were associated significantly with higher levels of job satisfaction, supporting Hypothesis 2. Additionally, supervisor support was associated negatively with emotional exhaustion, indicating that higher levels of supervisor support were associated with lower levels of emotional exhaustion. Workload and supervisor support explained 22% of the variance in emotional exhaustion and 11% of the variance in job satisfaction. To test the proposed mediation effects, we next entered WFC into the model as an intervening variable, and considered the partial mediation Model M_1_ (shown in **Figure 2**). Again, emotional exhaustion and job satisfaction were correlated through their error terms. The model fitted the data well, χ^2^(162) = 774.24, *p* = 0.000, CFI = 0.96, RMSEA = 0.06. Workload was associated positively and supervisor support was associated negatively with WFC. Together, they explained 13% of its variance. The ML estimate of the standardized indirect effect of workload on emotional exhaustion was positive and statistically significant (0.14, 95% bootstrap CI [0.10, 0.19], *p* = 0.006), indicating that higher levels of workload were related indirectly via an increase in WFC to higher levels of emotional exhaustion. Because the direct effect of workload on emotional exhaustion was still significant, results provided support for the partial mediation effect assumed in Hypothesis 3a. The ML estimate of the standardized indirect effect of supervisor support on job satisfaction was also positive and statistically significant [0.05, 95% bootstrap CI (0.03, 0.07), *p* = 0.009], indicating that higher levels of supervisor support were related indirectly via a reduction in WFC to higher levels in job satisfaction. This supported Hypothesis 3b. Again, results indicated a partial mediation effect, because of the significance of the direct effect of supervisor support on job satisfaction. In addition, the indirect effects not mentioned in our hypotheses, namely, the indirect negative effect of workload on job satisfaction (-0.06, 95% bootstrap CI [-0.09, -0.04], *p* = 0.005) and the indirect negative effect of supervisor support on emotional exhaustion [-0.11, 95% bootstrap CI (-0.15, -0.07), *p* = 0.009], were statistically significant.

We integrated the control variables job tenure, working full/part time, and type of shift work one at a time into the final Model M_1_ and considered their effects on study variables and model parameters. In line with the bivariate correlations reported in **Table 1** job tenure had significant effects on our study variables: workload (β = 0.15, *p* = 0.016) and emotional exhaustion (β = 0.16, *p* = 0.006) increased with increasing job tenure; supervisor support (β = -0.12, *p* = 0.009), WFC (β = -0.16, *p* = 0.009), and job satisfaction (β = -0.32, *p* = 0.004) decreased. Additionally, working part time was related to lower levels of WFC (β = -0.17, *p* = 0.009) and job satisfaction (β = -0.10, *p* = 0.018) and higher levels of emotional exhaustion (β = 0.06, *p* = 0.029) compared to working full time. Finally, working in fixed shifts or day duty significantly reduced WFC (β = -0.16, *p* = 0.007) and slightly increased emotional exhaustion (β = 0.05, *p* = 0.024) compared to working in changing shifts. However, the integration of job tenure, working full/part time, and type of shift work into M_1_ did not affect our major findings, namely, the direct and indirect effects of workload and supervisor support on emotional exhaustion and job satisfaction.

## Discussion

The aim of the present study was to explore the relationships between characteristics of the working environment (workload, supervisor support), emotional exhaustion, and job satisfaction among airport security officers (screeners). Emotional exhaustion and job satisfaction have proven to be important determinants of performance in general (e.g., Judge et al., 2001; Cropanzano et al., 2003; Bakker et al., 2004; Wright et al., 2007) and of safety performance in particular (Siu et al., 2004; Nahrgang et al., 2011). Despite the highly security-critical tasks of screeners, previous research has not focused on emotional exhaustion and job satisfaction in this important occupational group. The present study addressed this research gap by studying the effects of workload and supervisor support on emotional exhaustion and job satisfaction. The results of our study allow conclusions on which characteristics of screeners’ working environment affect emotional exhaustion and job satisfaction, and are thus crucial for the security concept at airports. Additionally, we investigated WFC as an intervening variable in the JD–R model and examined indirect effects of workload and supervisor support on emotional exhaustion and job satisfaction among screeners.

The results supported our hypotheses. Consistent with Hypotheses 1 and 2, workload and supervisor support were confirmed as antecedents of emotional exhaustion and job satisfaction in screeners. Our results are in line with the assumptions of the JD–R model (Bakker and Demerouti, 2007) and with research findings in other occupational settings demonstrating the crucial role of the relation between workload and emotional exhaustion (e.g., Sonnentag et al., 2010; van Ruysseveldt et al., 2011). Screeners may try to maintain performance standards despite a high workload. This, in turn, may lead to an extensive expenditure of emotional energy and eventually to emotional exhaustion (Cordes and Dougherty, 1993). Consistent with Hypothesis 2 and findings from research in other occupational settings (e.g., Lewig and Dollard, 2003; Cortese et al., 2010; Biggs et al., 2014) we confirmed supervisor support as a predictor of job satisfaction among screeners. Supervisor support may supply the basic human need for affiliation and, as a consequence, may boost job satisfaction, and thus foster strong mental health.

In accordance with Hypothesis 3a, results supported the assumption that WFC is an intervening variable in the relationship between workload and emotional exhaustion in screeners. This is in line with research findings in other occupational settings (Peeters et al., 2004; Demerouti et al., 2005; Hall et al., 2010). High workload may deplete emotional resources among screeners and consequently give rise to WFC (cf. Semmer et al., 2010). WFC, in turn, may boost the experience of strain and subsequently lead to emotional exhaustion through impaired recovery (Geurts et al., 2003).

Moreover, in line with our Hypothesis 3b, WFC partially mediated the effect of supervisor support on job satisfaction. Employees’ possibilities of gaining social support from their supervisor may well influence the motivational process, because supervisors may, directly or indirectly (e.g., through scheduling), support a family-friendly organization of work. This facilitates better adjustment and regulation and, in turn, reduces WFC. A good compatibility between family and work may, in turn, promote positive motivational outcomes such as high job satisfaction. These findings support and replicate recent empirical findings indicating the crucial role of supervisor support in WFC (Cortese et al., 2010; Muse and Pichler, 2011) and the important role of WFC in job satisfaction (Cortese et al., 2010; Amstad et al., 2011). Moreover, they broaden our knowledge on WFC in the stressor–strain chain by indicating a significant indirect effect of supervisor support on job satisfaction mediated via WFC. In previous research, this effect was either not tested statistically (e.g., Thompson et al., 2006; Yildirim and Aycan, 2008; Cortese et al., 2010; Lu et al., 2015) or non-significant (Ito and Brotheridge, 2012; Odle-Dusseau et al., 2012).

However, one could argue that the indirect effects could be considered as small, both in absolute terms and compared to the direct effects of workload and supervisor support on emotional exhaustion and job satisfaction. This is true, especially for the indirect effect of supervisor support on job satisfaction. However, this is not very surprising, because associations are often rather small in the social sciences and in non-experimental designs, and the product of these coefficients (i.e., the indirect effect) will, of course, be quite small as well (Berset et al., 2011). This indicates that many variables influence processes leading to emotional exhaustion and job satisfaction, including possible moderators (cf. Semmer et al., 1996). Therefore, we believe that our findings do enrich current research by indicating that WFC may play an additional role in the emergence of emotional exhaustion and job satisfaction among screeners. At the same time, the small indirect effect of supervisor support on job satisfaction highlights the need to explore other variables relating work and family as explanatory mechanisms in the motivational process of the JD–R model. For instance, recent empirical evidence suggests that work–family enrichment could be an intervening variable in the motivational process as well (Odle-Dusseau et al., 2012; Lu et al., 2015).

Workload, supervisor support, and WFC explained a considerable amount of variance in screeners’ emotional exhaustion (49%). This amount of explained variance is remarkable given the multifactorial conditionality of health- and well-being-related variables (Semmer et al., 1996). However, the amount of explained variance in job satisfaction was notably lower (15%). One possible explanation for this result is to be found in the medium-sized correlation between job satisfaction and emotional exhaustion. This substantial correlation between the dependent variables indicates that relevant amounts of variance in emotional exhaustion and job satisfaction may overlap; and, consequently, that a certain amount of common variance between the predictor variables and job satisfaction may be obscured by the substantial relations between the predictor variables and emotional exhaustion. Additionally, recent meta-analytic findings suggest that positive affect—as a major personality characteristic—is especially relevant in predicting affective (as compared to cognitive) job satisfaction (Kaplan et al., 2009). Because the measure of job satisfaction in the present study represents predominantly an assessment of affective job satisfaction, one can expect positive affect to be a major determinant, offering another explanation for the rather small effects of supervisor support and WFC on the respective measure of job satisfaction.

In addition to the effects assumed in Hypotheses 1 to 3, there were several significant crossover effects: (1) supervisor support was related directly to emotional exhaustion, indicating that the availability of social support from the supervisor leads to a direct decrease in emotional exhaustion. (2) Workload was related indirectly to job satisfaction, indicating that workload increases WFC, which, in turn, decreases job satisfaction. (3) Supervisor support was related indirectly to emotional exhaustion, indicating that supervisor support decreases WFC, which, in turn, increases emotional exhaustion. These crossover effects are in line with recent empirical evidence (e.g., Li et al., 2013; Goh et al., 2015) and indicate that it might not be suitable to strictly separate health-related and motivational processes when predicting emotional exhaustion and job satisfaction in screeners.

### Strengths, Limitations, and Suggestions for Future Research

The main strength of this study is the theoretically grounded approach of integrating WFC into the JD–R model as an intervening variable. Additionally, we based our analysis on a large sample of screeners, thereby enabling us to draw first reliable conclusions on which factors relate to emotional exhaustion and job satisfaction in this occupational setting. We hope that our study can set the stage for further investigations of aviation security, because well-being-related factors have not yet been the focus of research despite their relevance for performance and thus for the security of airports.

Our results revealed the crucial role of supervisor support in the job satisfaction of screeners. However, we considered only its direct effects. It will be particularly important for future research to investigate the moderating effect of supervisor support in the relationships between workload, emotional exhaustion, and WFC (cf. Luk and Shaffer, 2005; Karatepe, 2010). In addition, previous research has pointed to the complicated nature of support and the role it plays in burnout (Cordes and Dougherty, 1993). Future research could identify which specific aspects of work social support (i.e., support stemming from coworkers, supervisors, or the organization) result in a reduction of burnout and WFC and contribute to job satisfaction, and then extend this approach by including sources of support in private life. Additionally, it would be interesting for future research to explore positive processes at the work–home interface such as work–family enrichment or facilitation as levers in the motivational process of the JD–R model (cf. Odle-Dusseau et al., 2012; Lu et al., 2015). Finally, it would be valuable to examine specific job demands of screeners (e.g., the need for constant attention to the task, interactions with difficult passengers) as predictors of their emotional exhaustion.

Our study did have several limitations: first, the present findings were based on a sample taken from just one organization, and more research will be needed before they can be generalized. Second, results on the relations between variables were based on cross-sectional data. Therefore, we could make no causal inferences, and reversed causalities may well be possible. Indeed, especially in the context of WFC research, the possibility of reversed causalities or reciprocal relationships (i.e., loss spirals) seems very plausible (Demerouti et al., 2004; van der Heijden et al., 2008). Third, we relied on self-report data, and this may inflate the associations between variables through common method variance (Podsakoff et al., 2003). Nonetheless, Semmer et al. (1996) have pointed out that substantive associations between working conditions and health remain after controlling for common method variance. However, longitudinal studies and an integration of both observational and physiological measures will still be needed to further validate our study results. Finally, we used a single item as an indicator of workload. Future research should explore the relations between workload, WFC, and emotional exhaustion based on a reliable multi-item measure of workload.

### Practical and Theoretical Implications

Emotional exhaustion, job satisfaction, and the working conditions that influence them in screeners have not been a focus of research and health promotion in the past. Therefore, we hope that our study will set the stage for further investigations in this field. In view of the general need to promote the well-being and health of screeners along with the very specific need for aviation security, it is essential to engage in more research that can serve as a starting point for an appropriate health promotion of airport security staff.

From a practical perspective, our findings suggest that workload and supervisor support may play a crucial role in the emergence of emotional exhaustion and job satisfaction in screeners. They indicate that reducing the workload and promoting a supportive working environment may contribute to preventing emotional exhaustion and promoting job satisfaction. Moreover, results obtained in this study strengthen earlier findings suggesting that WFC can be a risk factor for mental health problems such as emotional exhaustion and for motivational correlates such as job satisfaction. However, for aviation security staff, no work–life balance initiatives have been implemented so far. Therefore, it will be essential for aviation security organizations to take WFC into account in future workplace health promotion in order to decrease the risk of emotional exhaustion among screeners, to enhance their job satisfaction, and, as a consequence, to decrease the risk of further serious health problems and adverse organizational and societal outcomes. Workload and supervisor support turned out to be promising staring points, because they proved to be associated with emotional exhaustion and job satisfaction both directly and through the effect of WFC. However, workload is often difficult to reduce at short notice. Therefore, to reinforce the motivational and inhibit the health–impairing process, it is probably just as important to invest in social support processes as it is to try to reduce job demands. Supervisor support seems to be particularly important here, because screeners work under conditions in which other job resources (e.g., autonomy) are typically low and other sources of recognition and support (e.g., coworkers, customers) are typically available only to a limited extent.

From a theoretical point of view, the extension of the JD–R model seems to be particularly important. Recent studies have called for research to shed more light on the processes connecting working conditions and health-related or motivational outcomes in the JD–R model (Demerouti and Bakker, 2011; Fernet et al., 2013). WFC represents a promising extension of the JD–R model that may improve our understanding of the processes leading to emotional exhaustion and job satisfaction. It will, therefore, be important to clearly distinguish characteristics of the working environment (i.e., job demands and job resources) from confrontational states (i.e., WFC) as a consequence of these psychosocial workplace characteristics and as determinants of further health-related and motivational outcomes.

## Author Contributions

All authors substantially contributed to the conceptualization of the manuscript as well as to the aquisition, analysis, and interpretation of data. All authors critically revised the content of the manuscript repeatedly and approved the final version to be published. All authors agreed to be accountable for all aspects of the work. SB as the leading author contributed to the development of the questionnaire, the aquisition, analysis, and interpretation of data. SB was responsible for the conceptualization and the writing of the manuscript. AK predominantly contributed to the development of the questionnaire, the aquisition and interpretation of data. AK repeatedly revised and refined the content of the manuscript critically. AS predominantly contributed to the development of the questionnaire, the aquisition and interpretation of data. AS repeatedly revised and refined the content of the manuscript critically.

## Conflict of Interest Statement

The authors declare that the research was conducted in the absence of any commercial or financial relationships that could be construed as a potential conflict of interest.

## Footnotes

1 Most importantly, hold baggage screening takes place at remote locations, in which screeners do not work in teams and are not exposed to passengers and the noise of airport security checkpoints.

## References

[B1] Alarcon G. M. (2011). A meta-analysis of burnout with job demands, resources, and attitudes. *J. Vocat. Behav.* 79 549–562. 10.1016/j.jvb.2011.03.007

[B2] Allen T. D. Herst D. E. Bruck C. S. Sutton M. (2000). Consequences associated with work-to-family conflict: a review and agenda for future research. *J. Occup. Health Psychol.* 5 278–308. 10.1037/1076-8998.5.2.278 10784291

[B3] Amstad F. T. Meier L. L. Fasel U. Elfering A. Semmer N. K. (2011). A meta-analysis of work-family conflict and various outcomes with a special emphasis on cross-domain versus matching-domain relations. *J. Occup. Health Psychol.* 16 151–169. 10.1037/a0022170 21280939

[B4] Ângelo R. P. Chambel M. J. (2014). The role of proactive coping in the job demands–resources model: a cross-section study with firefighters. *Eur. J. Work Organ. Psychol.* 23 203–216. 10.1080/1359432x.2012.728701

[B5] Angulo B. U. Osca A. (2012). Role stressors, task-oriented norm and job satisfaction: a longitudinal study. *J. Work Organ. Psychol.* 28 171–181.

[B6] Bakker A. B. Demerouti E. (2007). The job demands–resources model: State of the art. *J. Manag. Psychol.* 22 309–328. 10.1108/02683940710733115

[B7] Bakker A. B. Demerouti E. Dollard M. F. (2008). How job demands affect partners’ experience of exhaustion: Integrating work–family conflict and crossover theory. *J. Appl. Psychol.* 93 901–911. 10.1037/0021-9010.93.4.901 18642992

[B8] Bakker A. B. Demerouti E. Verbeke W. (2004). Using the job demands–resources model to predict burnout and performance. *Hum. Resour. Manage.* 43 83–104. 10.1002/hrm.20004

[B9] Bakker A. B. Geurts S. A. E. (2004). Toward a dual-process model of work–home interference. *Work Occupat.* 31 345–366. 10.1177/0730888404266349

[B10] Baruch-Feldman C. Brondolo E. Ben-Dayan D. Schwartz J. (2002). Sources of social support and burnout, job satisfaction, and productivity. *J. Occup. Health Psychol.* 7 84–93. 10.1037//1076-8998.7.1.84 11827236

[B11] Benda P. (2015). Commentary: harnessing advanced technology and process innovations to enhance aviation security. *J. Air Transp. Manag.* 48 23–25. 10.1016/j.jairtraman.2015.06.008

[B12] Berset M. Elfering A. Lüthy S. Lüthi S. Semmer N. K. (2011). Work stressors and impaired sleep: rumination as a mediator. *Stress Health* 27 e71–e82. 10.1002/smi.1337 27486625

[B13] Beutell N. J. (2010). Work schedule, work schedule control and satisfaction in relation to work-family conflict, work-family synergy, and domain satisfaction. *Career Dev. Int.* 15 501–518. 10.1108/13620431011075358

[B14] Beutell N. J. Schneer J. A. (2014). Work-family conflict and synergy among Hispanics. *J. Manag. Psychol.* 29 705–735. 10.1108/JMP-11-2012-0342

[B15] Biggs A. Brough P. Barbour J. P. (2014). Enhancing work-related attitudes and work engagement: a quasi-experimental study of the impact of an organizational intervention. *Int. J. Stress Manag.* 21 43–68. 10.1037/a0034508

[B16] Biggs A. T. Mitroff S. R. (2014). Improving the efficacy of security screening tasks: a review of visual search challenges and ways to mitigate their adverse effects. *Appl. Cogn. Psychol.* 29 142–148. 10.1002/acp.3083

[B17] Bolton L. M. R. Harvey R. D. Grawitch M. J. Barber L. K. (2012). Counterproductive work behaviours in response to emotional exhaustion: a moderated mediational approach. *Stress Health* 28 222–233. 10.1002/smi.1425 22281803

[B18] Borritz M. Kristensen T. S. (1999). *Copenhagen Burnout Inventory.* Copenhagen: National Institute of Occupational Health.

[B19] Borritz M. Rugulies R. Bjorner J. B. Villadsen E. Mikkelsen O. A. Kristensen T. S. (2006). Burnout among employees in human service work: design and baseline findings of the PUMA study. *Scand. J. Public Health* 34 49–58. 10.1080/14034940510032275 16449044

[B20] Bowling N. A. Alarcon G. M. Bragg C. B. Hartman M. J. (2015). A meta-analytic examination of the potential correlates and consequences of workload. *Work Stress* 29 95–113. 10.1080/02678373.2015.1033037

[B21] Bowling N. A. Eschleman K. J. Wang Q. (2010). A meta-analytic examination of the relationship between job satisfaction and subjective well-being. *J. Occup. Organ. Psychol.* 83 915–934. 10.1348/096317909x478557

[B22] Boyar S. L. Huang X. Xu N. (2014). The moderating impact of family role configurations. *Employ. Responsib. Rights J.* 26 115–133. 10.1007/s10672-013-9235-9

[B23] Browne M. Cudeck R. (1993). “Alternative ways of assessing equation model fit,” in *Testing Structural Equation Models*, eds Bollen K. A. Scott J. S. (Newbury Park, StateCA: Sage Publisher), 136–162.

[B24] Byron K. (2005). A meta-analytic review of work–family conflict and its antecedents. *J. Vocat. Behav.* 67 169–198. 10.1016/j.jvb.2004.08.009

[B25] Carlson D. S. Kacmar K. M. Wayne J. H. Grzywacz J. G. (2006). Measuring the positive side of the work–family interface: development and validation of a work–family enrichment scale. *J. Vocat. Behav.* 68 131–164. 10.1016/j.jvb.2005.02.002

[B26] Cohen J. (1988). *Statistical Power Analysis for the Behavioral Sciences*, 2nd Edn. Hillsdale, StateNJ: Erlbaum.

[B27] Cordes C. L. Dougherty T. W. (1993). A review and integration of research on job burnout. *Acad. Manage. Rev.* 18 621–656. 10.5465/amr.1993.9402210153

[B28] Cortese C. G. Colombo L. Ghislieri C. (2010). Determinants of nurses’ job satisfaction: the role of work–family conflict, job demand, emotional charge and social support. *J. Nurs. Manag.* 18 35–43. 10.1111/j.1365-2834.2009.01064.x 20465727

[B29] Crawford E. R. LePine J. A. Rich B. L. (2010). Linking job demands and resources to employee engagement and burnout: a theoretical extension and meta-analytic test. *J. Appl. Psychol.* 95 834–848. 10.1037/a0019364 20836586

[B30] Cropanzano R. Rupp D. E. Byrne Z. S. (2003). The relationship of emotional exhaustion to work attitudes, job performance, and organizational citizenship behaviors. *J. Appl. Psychol.* 88 160–169. 10.1037/0021-9010.88.1.160 12675403

[B31] Demerouti E. Bakker A. B. (2011). The job demands–resources model: challenges for future research. *SA J. Indust. Psychol.* 37 1–9. 10.4102/sajip.v37i2.974

[B32] Demerouti E. Bakker A. B. Bulters A. J. (2004). The loss spiral of work pressure, work–home interference and exhaustion: reciprocal relations in a three-wave study. *J. Vocat. Behav.* 64 131–149. 10.1016/s0001-8791(03)00030-7

[B33] Demerouti E. Bakker A. B. Schaufeli W. B. (2005). Spillover and crossover of exhaustion and life satisfaction among dual-earner parents. *J. Vocat. Behav.* 67 266–289. 10.1016/j.jvb.2004.07.001

[B34] Dormann C. Zapf D. (2001). Job satisfaction: a meta-analysis of stabilities. *J. Organ. Behav.* 22 483–504. 10.1002/job.98

[B35] Ducki A. (2000). *Diagnose Gesundheitsförderlicher Arbeit: Eine Gesamtstrategie zur betrieblichen Gesundheitsanalyse [Diagnosis of Health-Promoting Work].* Zürich: Hochschulverlag.

[B36] Fernet C. Austin S. Trépanier S.-G. Dussault M. (2013). How do job characteristics contribute to burnout? Exploring the distinct mediating roles of perceived autonomy, competence, and relatedness. *Eur. J. Work Organ. Psychol.* 22 123–137. 10.1080/1359432x.2011.632161

[B37] Fried Y. Shirom A. Gilboa S. Cooper C. L. (2008). The mediating effects of job satisfaction and propensity to leave on role stress–job performance relationships: combining meta-analysis and structural equation modeling. *Int. J. Stress Manag.* 15 305–328. 10.1037/a0013932

[B38] Frone M. R. Russell M. Cooper M. L. (1992). ). Antecedents and outcomes of work-family conflict: testing a model of the work-family interface. *J. Appl. Psychol.* 77 65–78. 10.1037/0021-9010.77.1.65 1556042

[B39] Frye N. K. Breaugh J. A. (2004). Family-friendly policies, supervisor support, work-family conflict, family-work conflict, and satisfaction: a test of a conceptual model. *J. Bus. Psychol.* 19 197–220. 10.1007/s10869-004-0548-4

[B40] Geurts S. A. E. Kompier M. A. J. Roxburgh S. Houtman I. L. D. (2003). Does work–home interference mediate the relationship between workload and well-being? *J. Vocat. Behav.* 63 532–559. 10.1016/s0001-8791(02)00025-8

[B41] Goh Z. Ilies R. Wilson K. S. (2015). Supportive supervisors improve employees’ daily lives: the role supervisors play in the impact of daily workload on life satisfaction via work–family conflict. *J. Vocat. Behav.* 89 65–73. 10.1016/j.jvb.2015.04.009

[B42] Guest D. E. (2002). Perspectives on the study of work-life balance. *Soc. Sci. Inform.* 41 255–279. 10.1177/0539018402041002005

[B43] Guglielmi D. Panari C. Simbula S. (2012). The determinants of teachers’ well-being. The mediating role of mental fatigue. *Eur. J. Ment. Health* 7 204–220. 10.5708/ejmh.7.2012.2.3

[B44] Hackman J. Oldham G. (1976). Motivation through the design of work: test of a theory. *Organ. Behav. Hum. Perform.* 16 250–279. 10.1016/0030-5073(76)90016-7

[B45] Hair J. F. Anderson R. E. Tatham R. L. Black W. (1998). *Multivariate Data Analysis.* Upper Saddle River, StateNJ: Prentice Hall.

[B46] Hair J. F. Black W. C. Babin B. J. Anderson R. E. (2014). *Multivariate Data Analysis (new int. ed.).* Harlow: Pearson Education.

[B47] Halbherr T. Schwaninger A. Budgell G. Wales A. (2013). Airport security screener competency: a cross-sectional and longitudinal analysis. *Int. J. Aviat. Psychol.* 23 113–129. 10.1080/10508414.2011.582455

[B48] Hall G. B. Dollard M. F. Tuckey M. R. Winefield A. H. Thompson B. M. (2010). Job demands, work–family conflict, and emotional exhaustion in police officers: a longitudinal test of competing theories. *J. Occup. Organ. Psychol.* 83 237–250. 10.1348/096317908x401723

[B49] Hellman C. M. (1997). Job satisfaction and intent to leave. *J. Soc. Psychol.* 137 677–689. 10.1080/00224549709595491

[B50] Hobfoll S. E. (1989). Conservation of resources. A new attempt at conceptualizing stress. *Am. Psychol.* 44 513–524. 10.1037//0003-066x.44.3.513 2648906

[B51] Hu L. Bentler P. M. (1999). Cutoff criteria fit indexes in covariance structure analysis: conventional criteria versus new alternatives. *Struct. Equ. Model.* 6 1–55. 10.1080/10705519909540118

[B52] Innstrand S. T. Langballe E. M. Espnes G. A. Falkum E. Gjerl O. (2008). Positive and negative work–family interaction and burnout: a longitudinal study of reciprocal relations. *Work Stress* 22 1–15. 10.1080/02678370801975842

[B53] Ironson G. H. Smith P. C. Brannik M. T. Gibson W. M. Paul K. B. (1989). Construction of a job in general scale: a comparison of global, composite, and specific measures. *J. Appl. Psychol.* 74 193–200. 10.1037/0021-9010.74.2.193

[B54] Ito J. K. Brotheridge C. M. (2012). Work-family and interpersonal conflict as levers in the resource/demand-outcome relationship. *Career Dev. Int.* 17 393–413. 10.1080/10615801003728273

[B55] Judge T. A. Thoresen C. J. Bono J. E. Patton G. K. (2001). The job satisfaction–job performance relationship: a qualitative and quantitative review. *Psychol. Bull.* 127 376–407. 10.1037//0033-2909.127.3.376 11393302

[B56] Kahn R. L. Wolfe D. M. Quinn R. P. Snoek J. D. Rosenthal R. A. (1964). *Organizational Stress: Studies in Role Conflict and Ambiguity.* New York, StateNY: Wiley.

[B57] Kaplan S. A. Warren C. R. Barsky A. P. Thoresen C. J. (2009). A note on the relationship between affect(ivity) and differing conceptualizations of job satisfaction: some unexpected meta-analytic findings. *Eur. J. Work Organ. Psychol.* 18 29–54. 10.1080/13594320701873264

[B58] Karasek R. A. (1979). Job demands, job decision latitude, and mental strain: implications for job redesign. *Admin. Sci. Quart.* 24 285–308. 10.2307/2392498

[B59] Karatepe O. M. (2010). The effect of positive and negative work-family interaction on exhaustion. Does work social support make a difference? *Int. J. Contemp. Hosp. M* 22 836–856. 10.1108/09596111011063115

[B60] Karatepe O. M. Kilic H. (2007). Relationships of supervisor support and conflicts in the work–family interface with the selected job outcomes of frontline employees. *Tourism Manage.* 28 238–252. 10.1016/j.tourman.2005.12.019

[B61] Karimbocus M. (2015). “Aviation security and organizational behavior,” in *Global Supply Chain Security*, eds Thomas A. R. Vaduva S. (New York, NY: Springer Science+Business Media), 81–97.

[B62] Kline R. B. (2011). *Principles and Practice of Structural Equation Modeling*, 3rd Edn. New York, StateNY: The Guilford Press.

[B63] Knudsen H. K. Ducharme L. J. Roman P. M. (2009). Turnover intention and emotional exhaustion “at the top”: adapting the job demands–resources model to leaders of addiction treatment organizations. *J. Occup. Health Psychol.* 14 84–95. 10.1037/a0013822 19210050 PMC2746447

[B64] Koller S. Drury C. Schwaninger A. (2009). Change of search time and non-search time in X-ray baggage screening due to training. *Ergonomics* 52 644–656. 10.1080/00140130802526935 19424926

[B65] Leavy R. L. (1983). Social support and psychological disorder: a review. *J. Commun. Psychol.* 11 3–21. 10.1002/1520-6629(198301)11 10259112

[B66] Lee R. T. Ashforth B. E. (1993). A further examination of managerial burnout: toward an integrated model of burnout. *J. Organ. Behav.* 14 3–20. 10.1002/job.4030140103

[B67] Lee R. T. Ashforth B. E. (1996). A meta-analytic examination of the correlates of the three dimensions of job burnout. *J. Appl. Psychol.* 81 123–133. 10.1037/0021-9010.81.2.123 8603909

[B68] Lewig K. A. Dollard M. F. (2003). Emotional dissonance, emotional exhaustion and job satisfaction in call centre workers. *Eur. J. Work Organ. Psychol.* 12 366–392. 10.1080/13594320344000200

[B69] Li F. Jiang L. Yao X. Li Y. (2013). Job demands, job resources and safety outcomes: the roles of emotional exhaustion and safety compliance. *Accident Anal. Prev.* 51 243–251. 10.1016/j.aap.2012.11.029 23274477

[B70] Locke E. A. (1976). “The nature and causes of job satisfaction,” in *Handbook of Industrial and Organizational Psychology*, ed. Dunnette M. D. (Chicago, StateIL: Rand McNally), 1297–1349.

[B71] Lu L. Chang T. Kao S. Cooper C. L. (2015). Testing an integrated model of the work–family interface in Chinese employees: a longitudinal study. *Asian J. Soc. Psychol.* 18 12–21. 10.1111/ajsp.12081

[B72] Luk D. M. Shaffer M. A. (2005). Work and family domain stressors and support: within- and cross-domain influences on work–family conflict. *J. Occup. Organ. Psychol.* 78 489–508. 10.1348/096317905x26741

[B73] MacKinnon D. P. Fairchild A. J. Fritz M. S. (2007). Mediation analysis. *Annu. Rev. Psychol.* 58 593–614. 10.1146/annurev.psych.58.110405.085542 16968208 PMC2819368

[B74] Maslach C. (1982). *Burnout: The Cost of Caring.* Englewood Cliffs, NJ: Prentice-Hall.

[B75] Mendes M. Schwaninger A. Michel S. (2013). Can laptops be left inside passenger bags if motion imaging is used in X-ray security screening? *Front. Hum. Neurosci.* 7:654 10.3389/fnhum.2013.00654 PMC3798983 24151457

[B76] Michel J. S. Kotrba L. M. Mitchelson J. K. Clark M. A. Baltes B. B. (2011). Antecedents of work–family conflict: a meta-analytic review. *J. Organ. Behav.* 32 689–725. 10.1002/job.695

[B77] Mitroff S. R. Biggs A. T. Cain M. S. (2015). Multiple-target visual search errors: overview and implications for airport security. *Policy Insights Behav. Brain Sci.* 2 121–128. 10.1177/2372732215601111

[B78] Muse L. A. Pichler S. (2011). A comparison of types of support for lower-skill workers: evidence for the importance of family supportive supervisors. *J. Vocat. Behav.* 79 653–666. 10.1016/j.jvb.2011.04.005

[B79] Nahrgang J. D. Morgeson F. P. Hofmann D. A. (2011). Safety at work: a meta-analytic investigation of the link between job demands, job resources, burnout, engagement, and safety outcomes. *J. Appl. Psychol.* 96 71–94. 10.1037/a0021484 21171732

[B80] Netemeyer R. G. Boles J. S. McMurrian R. (1996). Development and validation of work–family conflict and family–work conflict scales. *J. Appl. Psychol.* 81 400–410. 10.1037//0021-9010.81.4.400

[B81] Nübling M. Stössel U. Hasselhorn H. M. Michaelis M. Hofmann F. (2006). Measuring psychological stress and strain at work: evaluation of the COPSOQ questionnaire in Germany. *GMS Psycho Soc. Med.* 3 1–14. PMC2736502 19742072

[B82] Nunnally J. C. Bernstein I. H. (1994). *Psychometric Theory*, 3rd Edn. New York, StateNY: McGraw-Hill.

[B83] Odle-Dusseau H. N. Britt T. W. Greene-Shortridge T. M. (2012). Organizational work–family resources as predictors of job performance and attitudes: the process of work–family conflict and enrichment. *J. Occup. Health Psychol.* 17 28–40. 10.1037/a0026428 22149204

[B84] Parker P. A. Kulik J. A. (1995). Burnout, self- and supervisor-rated job performance, and absenteeism among nurses. *J. Behav. Med.* 18 581–599. 10.1007/bf01857897 8749987

[B85] Pedersen D. E. Minnotte K. L. (2012). Self- and spouse-reported work–family conflict and dual-earners’ job satisfaction. *Marriage Fam. Rev.* 48 272–292. 10.1080/01494929.2012.665015

[B86] Peeters M. C. W. de Jonge J. Janssen P. P. M. van der Linden S. (2004). Work–home interference, job stressors, and employee health in a longitudinal perspective. *Int. J. Stress Manag.* 11 305–322. 10.1037/1072-5245.11.4.305

[B87] Peeters M. C. W. Montgomery A. J. Bakker A. B. Schaufeli W. B. (2005). Balancing work and home: how job and home demands are related to burnout. *Int. J. Stress Manag.* 12 43–61. 10.1037/1072-5245.12.1.43

[B88] Petrou P. Demerouti E. Peeters M. C. W. Schaufeli W. B. Hetland J. (2012). Crafting a job on a daily basis: Contextual correlates and the link to work engagement. *J. Organ. Behav.* 33 1120–1141. 10.1002/job.1783

[B89] Podsakoff P. M. MacKenzie S. B. Lee J. Podsakoff N. P. (2003). Common method biases in behavioral research: a critical review of the literature and recommended remedies. *J. Appl. Psychol.* 88 879–903. 10.1037/0021-9010.88.5.879 14516251

[B90] Rimann M. Udris I. (1997). “Subjektive arbeitsanalyse: der fragebogen SALSA” [Subjective work analysis: the SALSA questionnaire],” in *Unternehmen Arbeitspsychologisch Bewerten. Ein Mehrebenen-Ansatz Unter Besonderer Berücksichtigung von Mensch, Technik und Organisation*, eds Strohm U. Ulich E. (Zürich: Hochschulverlag), 281–298.

[B91] Rödel A. Siegrist J. Hessel A. Brähler E. (2004). Fragebogen zur Messung beruflicher Gratifikationskrisen [Questionnaire to measure effort–reward imbalance at work]. *Z. Differ. Diagnost. Psychol.* 25 227–238. 10.1024/0170-1789.25.4.227

[B92] Schaufeli W. B. Bakker A. B. Van Rhenen W. (2009). How changes in job demands and resources predict burnout, work engagement and sickness absenteeism. *J. Organ. Behav.* 30 893–917. 10.1002/job.595

[B93] Semmer N. K. Grebner S. Elfering A. (2010). “Psychische Kosten von Arbeit: Beanspruchung und Erholung, Leistung und Gesundheit” [Mental costs of work: Strain and recovery, performance, and health],” in *Enzyklopädie der Psychologie: Themenbereich D Praxisgebiete, Serie III Wirtschafts-, Organisations- und Arbeitspsychologie, Band 1 Arbeitspsychologie*, eds Kleinbeck U. Schmidt K.-H. (Göttingen: Hogrefe), 325–370.

[B94] Semmer N. K. Zapf D. Greif S. (1996). Shared job strain: a new approach for assessing the validity of job stress measurements. *J. Occup. Organ. Psychol.* 69 293–310. 10.1111/j.2044-8325.1996.tb00616.x

[B95] Siegrist J. (1996). Adverse health effects of high-effort/low-reward conditions. *J. Occup. Health Psychol.* 1 27–41. 10.1037/1076-8998.1.1.27 9547031

[B96] Siegrist J. Starke D. Chandola T. Godin I. Marmot M. Niedhammer I. (2004). The measurement of effort–reward imbalance at work: European comparisons. *Soc. Sci. Med.* 58 1483–1499. 10.1016/s0277-9536(03)00351-4 14759692

[B97] Siu O. Phillips D. R. Leung T. (2004). Safety climate and safety performance among construction workers in Hong Kong. The role of psychological strains as mediators. *Accident Anal. Prev.* 36 359–366. 10.1016/s0001-4575(03)00016-2 15003580

[B98] Snow D. L. Swan S. C. Raghavan C. Connell C. M. Klein I. (2003). The relationship of work stressors, coping, and social support to psychological symptoms among female secretarial employees. *Work Stress* 17 241–263. 10.1080/02678370310001625630

[B99] Sonnentag S. Kuttler I. Fritz C. (2010). Job stressors, emotional exhaustion, and need for recovery: a multi-source study on the benefits of psychological detachment. *J. Vocat. Behav.* 76 355–365. 10.1016/j.jvb.2009.06.005

[B100] Thompson B. M. Brough P. A. Schmidt H. (2006). Supervisor and subordinate work–family values: does similarity make a difference? *Int. J. Stress Manag.* 13 45–63. 10.1037/1072-5245.13.1.45

[B101] van der Heijden B. Demerouti E. Bakker A. B. Hasselhorn H.-M. (2008). Work-home interference among nurses: Reciprocal relationships with job demands and health. *J. Adv. Nurs.* 62 572–584. 10.1111/j.1365-2648.2008.04630.x 18489450

[B102] van Ruysseveldt J. Verboon P. Smulders P. (2011). Job resources and emotional exhaustion: the mediating role of learning opportunities. *Work Stress* 25 205–223. 10.1080/02678373.2011.613223

[B103] van Woerkom M. Bakker A. B. Nishii L. H. (2016). Accumulative job demands and support for strength use: fine-tuning the job demands-resources model using conservation of resources theory. *J. Appl. Psychol.* 101 141–150. 10.1037/apl0000033 26121090

[B104] von Bastian C. Schwaninger A. Michel S. (2008). Do multi-view X-ray systems improve X-ray image interpretation in airport security screening? *Z. Arbeitswissens.* 3 166–173.

[B105] Voydanoff P. (2002). Linkages between the work-family interface and work, family, and individual outcomes. An integrative model. *J. Fam. Issues* 23 138–164. 10.1177/0192513x02023001007

[B106] Wetter O. E. (2013). Imaging in airport security: past, present, future, and the link to forensic and clinical radiology. *J. For. Radiol. Imag.* 1 152–160. 10.1016/j.jofri.2013.07.002

[B107] Wolfe J. M. Brunelli D. N. Rubinstein J. Horowitz T. S. (2013). Prevalence effects in newly trained airport checkpoint screeners: trained observers miss rare targets, too. *J. Vis.* 13:33 10.1167/13.3.33 PMC3848386 24297778

[B108] Wright T. A. Bonett D. G. (1997). The contribution of burnout to work performance. *J. Organ. Behav.* 18 491–499. 10.1002/(sici)1099-1379(199709)18

[B109] Wright T. A. Cropanzano R. (1998). Emotional exhaustion as a predictor of job performance and voluntary turnover. *J. Appl. Psychol.* 83 486–493. 10.1037//0021-9010.83.3.486 9648526

[B110] Wright T. A. Cropanzano R. Bonett D. G. (2007). The moderating role of employee positive well-being on the relation between job satisfaction and job performance. *J. Occup. Health Psychol.* 12 93–104. 10.1037/1076-8998.12.2.93 17469992

[B111] Xanthopoulou D. Bakker A. B. Demerouti E. Schaufeli W. B. (2007). The role of personal resources in the job demands-resources model. *Int. J. Stress Manag* 14 121–141. 10.1037/1072-5245.14.2.121

[B112] Yildirim D. Aycan Z. (2008). Nurses’ work demands and work–family conflict: a questionnaire survey. *Int. J. Nurs. Stud.* 45 1366–1378. 10.1016/j.ijnurstu.2007.10.010 18262529

